# Moderate–Vigorous Physical Activity, Screen Time and Sleep Time Profiles: A Cluster Analysis in Spanish Adolescents

**DOI:** 10.3390/ijerph20032004

**Published:** 2023-01-21

**Authors:** Daniel Sanz-Martín, Félix Zurita-Ortega, Germán Ruiz-Tendero, José Luis Ubago-Jiménez

**Affiliations:** 1 Department of Didactics of Musical, Plastic and Corporal Expression, Faculty of Humanities and Educational Sciences, University of Jaén, 23071 Jaén, Spain; 2 Department of Didactics Musical, Plastic and Corporal Expression, Faculty of Education Science, University of Granada, 18071 Granada, Spain; 3 Department of Languages, Arts and Physical Education Teaching, Faculty of Education, Complutense University of Madrid, 28040 Madrid, Spain

**Keywords:** physical activity, screen time, sleep time, adolescents

## Abstract

The study had two aims: (1) To classify the adolescents according to their levels of moderate–vigorous physical activity, screen time and sleep time, and (2) to analyze, in a descriptive and correlational manner, the profiles of moderate–vigorous physical activity, screen time and sleep time of each cluster according to the sex and grade of the adolescents. The study design was cross-sectional, with descriptive and correlational analysis. The sample consisted of 663 adolescents in Compulsory Secondary Education from Soria (Spain). The Four by One-Day Physical Activity Questionnaire was used to measure levels of physical activity, screen time and sleep time. The results showed that the young people had an average of 67.99 ± min/day of moderate–vigorous physical activity, 112.56 min/day of screen time and 548.63 min/day of sleep time. Adolescents were classified into three clusters according to their levels of physical activity, screen time and sleep time (F_MANOVA_ (6,1318) = 314.439; *p* ≤ 0.001; β = 1; f = 1.177). In conclusion, only 28.21% of the young people accomplished the recommendations for physical activity practice, screen time and sleep time. Moreover, these results vary according to the sex and grade of the adolescents.

## 1. Introduction

Preventable premature deaths account for 3.9 million deaths annually, which currently represents 15% of deaths [1]. This percentage of deaths varies by territorial region and has declined substantially since 1990 [2]. One of the main causes of premature death is physical inactivity, which accounts for 6.4% of these deaths [1]. In addition to health problems, physical inactivity also involves global economic problems, such as costs of 53.8 billion dollars to healthcare systems and 13.7 billion dollars in lost productivity as a result of deaths from physical inactivity [3]. Another cause of premature death is obesity [4]. It is estimated that more than 340 million children and adolescents suffer from overweight and obesity [5], and these levels continue to increase [6]. Sedentary lifestyles are one of the main causes of high levels of overweight and obesity [6,7].

Physical activity practice not only helps prevent overweight/obesity and preventable premature death but also has numerous physiological, psychological and social benefits [8,9]. Some benefits of physical activity in children and adolescents are improved cardiometabolic health, bone mass and cognitive functioning, the reduced risk of depression, and it helps to maintain healthy fitness and weight [10,11,12]. For physical activity to be healthy, it should be performed according to international recommendations [13]. The World Health Organization [10] and the U.S. Department of Health and Human Services [14] advise that children and adolescents should engage in at least 60 min a day of moderate-to-vigorous physical activity, mainly aerobic, as well as bone-strengthening activities, at least three days a week.

Despite the scientific evidence regarding the health problems described above, characterized by high rates of preventable premature death and overweight/obesity, and the benefits derived from physical activity, the levels of physical inactivity among adolescents are very high. According to the study by Guthold et al. [15], 81% of young people worldwide aged 11 to 17 years old are inactive, i.e., they do not meet physical activity recommendations. At the European level, Steene-Johannessen et al. [16] showed that 71% of young people aged 10 to 18 years were inactive. In Spain, Santos-Labrador [17] found that 82% of adolescents were inactive, Sanz-Martín [18] showed that 86.6% were inactive, and Castañeda-Vázquez [19] found that 89.8% were inactive.

There are numerous determinants that influence physical activity levels in young people, such as sex (higher levels in males), age (levels decrease with increasing age) [20], sedentary behaviors after school [21] and sleep time [22]. Despite this widespread evidence, it is necessary to identify the physical activity levels of each population group and the specific influence correlations in order to design more effective priority proposals to reverse high levels of physical inactivity [15,23]. Furthermore, the in-depth study of physical activity levels and their determinants is especially important in adolescence since this is a sensitive phase for acquiring and consolidating healthy habits [18].

Adolescents engage in sedentary activities during their free time, such as through screen-based activities (e.g., playing video games, on computers or watching TV) [24]. Such is the acceptance of screen-based activities by adolescents that they often exceed the WHO recommended maximum time of 2 h/day [25,26,27,28,29]. Moreover, the relationship between physical activity and screen time is negative [21,30,31]. Likewise, the older adolescents are, the more likely they are to engage in sedentary activities and fewer physical activities, partially justifying, in this way, the increase in body fat, the reason for increasing rates of overweight and obesity worldwide [32]. Although the problems of high physical inactivity and excessive screen time of adolescents are not just a current issue, Ghekiere et al. [33] showed that in 2014, European adolescents were more active than those in 2002, but they also performed more screen time activity.

In addition to physical activity and screen time, another lifestyle determinant is sleep time. Current international guidelines establish that children aged 5 to 13 years should sleep at least 9 h per day and that adolescents aged 14 to 17 years should sleep at least 8 h [34,35]. Spanish adolescents sleep an average of 8 h and 28 min on school days and 9 h and 41 min on weekend days [36]. Adolescents’ compliance with sleep time recommendations is positively and significantly related to compliance with physical activity and screen time [22].

Tapia-Serrano et al. [37] found that only 7.12% of young people (preschoolers to adolescents) worldwide met international recommendations for physical activity, screen time and sleep time. This implies that all three lifestyle habits need to be considered at the same level of attention and promoted equally [38].

As a consequence of all the above, it is considered important to know the state of the art of adolescents in the province of Soria (Spain) according to different profiles in order to design, if necessary, more precise and effective health promotion actions. Therefore, the following research aims were established: (1) To classify adolescents according to their levels of moderate–vigorous physical activity, screen time and sleep time, and (2) to analyze, descriptively and correlationally, the profiles of moderate–vigorous physical activity, screen time and sleep time of each cluster according to the sex and grade of the adolescents.

## 2. Materials and Methods

### 2.1. Design and Subjects

The study is contextualized in the behavioral epidemiology paradigm [39], and the method used was cross-sectional, with a descriptive and correlational analysis of the physical activity levels, screen time and sleep time of adolescents [40].

The research was carried out in Soria, a disadvantageous area of Spain. It is composed of 183 municipalities, 171 of which comprise less than 1000 inhabitants. The population is 89,041 inhabitants, and its population density is 8.64 inhabitants/km^2^. It is also the Spanish region with the third-lowest GDP [41].

The study population consisted of 3224 adolescents living in Soria (Spain) and studying Compulsory Secondary Education (12–17 years). A non-probabilistic convenience sampling was carried out based on the criterion of accessibility so that the groups of students selected from each school could answer the questionnaire on the same days of administration. In addition, 17 of the 19 schools in Soria decided to participate.

The final sample was 663 adolescents, which represents a precision error of 3.39% for a standard deviation of 50 and a confidence level of 95%. The average age of the sample was 14.05 years (±1.26). Table 1 shows the descriptive characteristics of the adolescent sample.

### 2.2. Instrument and Variables

The instrument used in the research was the Four by One-Day Physical Activity Questionnaire (FBODPAQ), which calculates physical activity, screen time and sleep time variables. This questionnaire was initially designed and validated by Cale [42] for British adolescents. It was subsequently adapted and validated by Soler et al. [43] for Spanish adolescents, with a Cronbach’s alpha reliability value of α = 0.832. This questionnaire has been used in previous studies to assess the physical activity levels of adolescents [13,18,44,45,46].

FBODPAQ is administered across four days and asks about the physical activity performed by the adolescents on the previous day (except for Saturday, which is asked on the following Monday). One of the days is administered to find out the physical activity carried out on a school day when physical education was performed, another time for a school day when physical education was not performed, a third day is asked about the physical activity performed on a Saturday and another about the physical activity performed on a Sunday. The questionnaire has two formats, one for school days and one for weekend days. The school-day format is segmented into two parts: morning (differentiated into: before class, during class breaks and at lunchtime) and afternoon–evening. The weekend questionnaire format differentiates between morning, afternoon and evening.

The moderate–vigorous physical activity variable is measured in time of practice (minutes/day) and is calculated as the daily average of the sum of the results of all the items that ask about the time of physical activity associated with an average energy expenditure of at least 4 METs/hour, as established in the questionnaire protocol.

The screen time variable is measured in minutes/day and is also calculated as the daily average of the sum of time spent watching television and using computers, video games and the Internet. The sleeping time variable is calculated by subtracting from 24 h of the day the time that the adolescents respond that they have spent awake (time between waking up and going to bed). This variable is also expressed as average minutes/day.

### 2.3. Procedure

The study began with an in-depth search relating to the research topic. Afterward, the research project was designed, and the necessary permissions to carry it out were requested. The research respected the principles of the Declaration of Helsinki and was approved by the Ethics Committee of the University of Granada (1478/CEIH/2020). In addition, permissions were obtained from the head of the Provincial Directorate of Education of Soria and from the directors of the educational centers. Likewise, an informed consent form was provided to all of the selected participants, who had to return it signed by their legal guardians. Subsequently, the physical activity questionnaire was administered to the adolescents in their respective schools. As established in the protocol, this questionnaire was administered in a paper format, and there was one interviewer for every six respondents. The average response time to the questionnaire was approximately fifteen minutes. Finally, data analysis was performed, and the final report was drafted.

### 2.4. Data Analysis

IBM SPP 26.0 software (International Business Machines Corporation, Armonk, NY, USA) was used for the statistical analysis. The data were preliminarily cleaned, and no missing values were found. In addition, as the variables were expressed in different measurement ranges, they were typified (z-scores) in order to perform the cluster analysis [47]. Thirty-one outliers (greater than 3.0 or less than −3.0) were obtained, so these participants were eliminated in order not to distort the results of the following phases [48]. The final sample was 663 participants. Afterward, a descriptive statistical analysis of the variables was performed, including the Kolmogorov–Smirnov test for normality, skewness and kurtosis analyses. The average values of the variables were compared according to the sex of the participants using Student’s t-tests (independent samples) and Mann–Whitney U tests (two samples). ANOVA test (one factor), with Tukey’s contrast analysis and Kruskal–Wallis H test, were used to make the comparison according to the students’ grades. Subsequently, Pearson’s and Spearman’s correlations were calculated between the variables, depending on whether or not the variables followed the normal distribution, respectively.

The main analysis was performed following the phases established by Hair et al. [48], in line with the research aims. The first phase grouped the processes aimed at classifying the participants into clusters according to their values on the variables moderate–vigorous physical activity, screen time and sleep time. For this purpose, Ward’s hierarchical agglomerative and the non-hierarchical k-means procedure were applied together, which is common in social research [47]. Ward’s method, based on the squared Euclidean distance, facilitated the identification of the optimal number of clusters and the identification of outlier cases. This method minimizes the inter-subject distance within the cluster and avoids the creation of long chains [49]. Subsequently, the k-means method made it possible to classify the cases in the clusters defined in the previous process.

The second phase of the main analysis served to corroborate that there were significant differences between the study variables in the different clusters obtained. For this, the MANOVA test was used for one independent variable (cluster membership) and three dependent variables (moderate–vigorous physical activity, screen time and sleep time). In this way, the classification was validated. The Pillai trace test was used to identify the effect between the variables [50]. In addition, Levene’s test for equality of error variances was calculated. Based on the result of Levene’s test, Tukey’s post hoc data were selected (Levene’s *p*-value ≤ 0.05). Finally, the Kolmogorov–Smirnov test was performed for each variable in each cluster, and the Pearson’s and Spearman’s correlations between variables were calculated.

## 3. Results

The results showed that the average moderate–vigorous physical activity time of the adolescents was 67.99 ± 43.51 min, the average screen time was 112.56 ± 65.80 min, and the average sleeping time was 548.63 ± 50.21 min. Table 2 shows the descriptive statistics of the variables according to the sex and grade of the students.

Table 3 shows the descriptive z-scores of the variables for a 95% confidence interval, normal distribution, skewness, kurtosis and Pearson/Spearman correlations. The variables of moderate–vigorous physical activity and screen time follow a normal distribution, but sleeping time does not.

Based on the above, significant differences were obtained as a function of sex in moderate–vigorous physical activity (Levene F = 11.175 *p* = 0.001; t (654.92) = 7.3 *p* ≤ 0.001), in screen time (Levene F = 5.79 *p* = 0.016; t (659.23) = 7.19 *p* ≤ 0.001) and in sleep time (U = 37377.5 *p* ≤ 0.001). Significant differences depending on the grade were also obtained in the variables of moderate–vigorous physical activity (Levene F (3659) = 7.44 *p* ≤ 0.001; ANOVA F = 9.72 *p* ≤ 0.001) and screen time (Levene F (3659) = 5.79 *p* = 0.004; ANOVA F = 3.16 *p* = 0.024). Table 4 shows the multiple comparisons of the variables according to the students’ grades.

Regarding the comparison of sleeping time and student grade, significant differences were also obtained (H = 53.95 *p* ≤ 0.001). In addition, the differences were significant at a level of *p* ≤ 0.001, comparing the sleeping time of first- and third-grade students (x^2^ = 100.54), first- and fourth-grade students (x^2^ = 145) and second- and fourth-grade students (x^2^ = 97.81), and at a level *p* ≤ 0.05 comparing the rest of the grades (1st–2nd: x^2^ = 47.19; 2nd–3rd: x^2^ = 53.35; 3rd–4th: x^2^ = 44.46).

It was decided to choose three clusters based on the results obtained in Ward’s method, specifically those relating to the squared Euclidean distance proximity matrix, the clustering history and the dendrogram. Descriptive statistics of the number of potential clusters were also taken into account. Table 5 shows the results of the k-means classification based on the three stipulated clusters.

**Figure 1:**
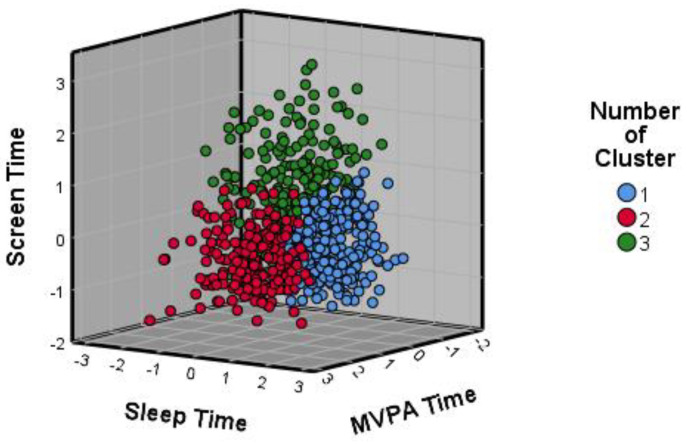
The scatter plot of participants’ z-scores. Note. Moderate–vigorous Physical Activity (MVPA).

Figure 1 shows the scatter plot of the cases as a function of the three study variables and the cluster of membership.

The values of the sleeping time variable in each of the clusters follow normal distributions according to the Kolmogorov–Smirnov test (*p* > 0.05), as well as the screen time in cluster 3. In addition, the values of the skewness ranged from −0.112 to 0.823 and those of kurtosis from −0.530 to 0.533.

The results obtained from the MANOVA test were used to validate the classification of three clusters. The values of the Pillai trace test were: F(6,1318) = 314.439; *p* ≤ 0.001; β = 1; f = 1.177.

The results of the Levene test for moderate–vigorous physical activity (F(2660) = 6.406), screen time (F(2660) = 29.626) and sleep time (F(2660) = 3.812) were not significant (*p* > 0.05), so Tukey’s post hoc was selected assuming equality of variances. Table 6 shows the multiple comparisons between the different clusters according to each variable.

Finally, Pearson and Spearman correlations were calculated between the variables in each of the clusters (Table 7). These correlations are positive and mostly significant and weak (0.10 < r < 0.30).

## 4. Discussion

In the present study, the two established aims were met. In relation to the first aim, the adolescents of the province of Soria were classified according to the level of moderate–vigorous physical activity, screen time and sleeping time. Specifically, the MANOVA test validated the identification of the three groups of the cluster analysis. With regard to the second aim, the profiles of moderate–vigorous physical activity, screen time and sleeping time of each cluster were analyzed descriptively and correlationally according to the sex and grade of the adolescents.

The average values of moderate–vigorous physical activity of the participants were higher than the 60 min/day recommended by the WHO [5] and the U.S. Department of Health and Human Services [14] and those found in other studies [51,52,53,54]. Screen time was lower than the maximum recommended 120 min/day by the WHO [10] and the findings of other studies [25,26,27,28,29,37]. Sleeping time was higher than the minimum recommended for their age [34,35] and those shown in other studies [33,36,37,55] but lower by almost three minutes (551 ± 54 min/day) than in the study by Peiró-Velert et al. in Spanish adolescents [45]. According to the sex of the participants, males obtained higher levels of physical activity and screen time. As a function of grade, physical activity and sleep time decreased from 1st to 4th grade, and screen time increased. These differences according to sex and grade are in line with what is established in the scientific literature [20,37,38,53,56].

In the clusters identified, the results found for the different variables vary. The participants in the first cluster did not accomplish the recommended moderate–vigorous physical activity time, but they did meet the recommended screen time and sleep time. The results for moderate–vigorous physical activity time may be due to the fact that the majority of the participants were females, being also the cluster with the highest percentage. This is related to what is established in the scientific literature regarding females obtaining lower levels of physical activity than males [20,37,53]. The average time spent sleeping is the highest of the three clusters and may be due to the fact that it is the cluster with the lowest representation of senior students. This is related to Chaput et al. [38] and Li [56], who showed that sleep time decreases with age.

The average levels of physical activity, screen time and sleep time of the second cluster are in line with international recommendations on these variables. In addition, the time spent in moderate–vigorous physical activity is almost double the recommended time (119.41 min). This cluster represents 28.21% of the total participants. The compliance with the recommendations may be due to the fact that most of the young people in the cluster were male and that almost one-third of the participants were first graders and another third were second graders [20,37,38,53,56].

The participants in the third cluster only met the recommendation for sleep time, although the value was the lowest of the three clusters. The average value for moderate–vigorous physical activity time is the lowest of all the groups, and screen time is the highest, exceeding the maximum recommended time by almost an hour. These results may be largely due to the fact that 62.4% of the students attend the two upper grades, and 37.2% of them attend the upper grade [20,37,38,53,56].

The study found different correlations between the research variables in each of the three clusters. In the first cluster, there were positive, weak and significant correlations between sleeping time, moderate–vigorous physical activity and screen time. In the second cluster, all three correlations were positive and significant, with weak correlations between screen time and moderate–vigorous physical activity and between screen time and sleep time. In addition, there was a moderate correlation between moderate–vigorous physical activity and sleeping time. In the third cluster, the correlation between screen time and sleeping time was positive, moderate and significant.

Other previous studies [22,57] also found the same trend in the correlations between sleeping time and physical activity and between sleeping time and screen time. On the other hand, regarding the relationship between physical activity and screen time, the trend found in previous studies is different, being negative [21,30]. This difference may be due to the fact that the relationship between physical activity varies as a function of screen time activity time. In the study conducted in Soria, screen time encompassed the time that young people spent “watching TV” and “using computers, video games and the Internet”, but not so in the other studies. Bejarano et al. [30] calculated the time spent watching television, videos, or DVDs and found a negative correlation between this time and physical activity in both boys and girls. In contrast, Braig et al. [31] found a positive relationship between TV viewing and PA but a negative relationship with other types of screen activity in 13-year-olds.

In the present study, it has been shown that sleeping time has special importance in healthy habits. The average time spent sleeping by adolescents in Soria was higher than recommended [34,35] and higher than that found in other studies [33,36,37,55]. In addition, the average time spent sleeping by young people in the three groups was also higher than recommended, with significant differences between all groups. Likewise, there were positive and significant correlations between sleep time and the other variables in all three clusters, with the exception of the relationship with physical activity in the third cluster. These sleep time results could have numerous benefits for the young people of Soria [58,59,60], such as the maintenance of cognitive function, attention, reaction time, working memory, visual–motor performance, decision-making, verbal function and motivation [61].

Based on the above, this study highlights the importance of the sex and age of adolescents in their healthy habits. Two of the causes that could justify the differences according to sex are the existence of sex stereotypes and the perception of barriers linked to the practice of physical activity. In relation to stereotypes, Fernández et al. [62] provide that there are two sets of traits: instrumental (related to masculinity) and affective–expressive (related to femininity). In turn, the instrumental ones are related to higher levels of physical activity. In addition, the existence of stereotypes among adolescents increases with age [63]. Regarding barriers to physical activity, Serra et al. [64] found that Spanish adolescents cited a lack of time as the main cause. In addition, females obtained higher scores in “not having time”, “having a lot of homework” and “studying a lot”.

There are several factors to consider in the influence of the age of adolescents on their healthy habits. One of these factors could be common to different geographic areas since adolescents in higher grades highlight academic demands as one of the barriers [64]. Another factor could be specific to Soria. The difference in levels of physical activity and screen time could be partially due to the size of the municipalities (93.44% have less than 1000 inhabitants), the high average age of the population (47.67 years) and the high percentage of the population over 60 years (28.72%) [41]. This would imply that young people in Soria would find it difficult to engage in physical activity, as it has been shown that the direct relationship with peers has a substantial influence [18]. In addition, only ten municipalities of the 183 existing in Soria have educational centers that offer Compulsory Secondary Education, which means that many adolescents have to spend daily time going to and from the centers.

In relation to the results found and to those provided by Guthold et al. [15] and Lizandra et al. [23], it would be advisable to make proposals to improve the healthy habits of adolescents in Soria based on the profiles of the clusters. To this, actions should be prioritized in the participants of cluster three since they do not meet the recommendations for physical activity and exceed those for screen time by almost an hour. With the participants in cluster two, the focus should be on increasing the time spent performing physical activity. Despite prioritizing these actions, other proposals should be made to consolidate the habits in which the recommendations are met. Likewise, emphasis should be placed on the healthy habits of females and students in higher grades.

The study conducted had some limitations. There was a limitation associated with the instrument used. Questionnaires have been and will be widely used in scientific research, as they have numerous advantages but also some drawbacks. One of these drawbacks is that they are subjective in nature, with participants answering according to their own considerations. This means that they are not as accurate as other instruments, such as accelerometers, for measuring physical activity. However, it should be taken into account that the questionnaire used has been previously validated and that it allows a larger number of participants to be selected. Another limitation was that only night-time rest was asked, as established in the questionnaire protocol, but “siesta” was not taken into account. The last limitation was that the activities that included screen time were “watching TV” and “using computers, video games and the Internet”. Perhaps the results would have been different if other activities, such as mobile phone/tablet use, had been included.

It would be advisable to conduct future research on the topic studied. It would be very interesting to carry out a longitudinal study to determine how the levels of moderate–vigorous physical activity time, screen time, and sleep time of adolescents in Soria vary over time and the correlations between these variables. A study could also be conducted by expanding the number of participants and including people of all ages. Another study could include other healthy habits, such as nutrition and hygiene. In addition, proposals should be designed to improve the levels of physical activity, reduce screen time and maintain the sleep time of adolescents in Soria, prioritizing actions based on the profiles of the clusters so that they are more effective. For example, it would be convenient to consider designing proposals to improve physical activity during recess time in educational centers [65].

## 5. Conclusions

Finally, the conclusions will be mentioned. The average values of moderate–vigorous physical activity, screen time and sleeping time of adolescents in Soria meet international recommendations. These results vary according to sex and grade, being higher in males than in females and in first-year students than in fourth-year students.

The classification of adolescents into three clusters according to their levels of moderate–vigorous physical activity, time spent at school and time spent sleeping has been shown to be valid. This classification can be useful for designing more contextualized and effective health promotion proposals.

According to the average values of the variables, the young people in cluster 1 meet the international recommendations for screen time and sleeping time but not for physical activity. In this group, there are positive, slight and significant relationships between physical activity, sleeping time and screen time. The adolescents in the second cluster meet all the recommendations. In addition, the relationships between the three healthy variables are positive, significant and predominantly slight. The members of the third cluster only meet the recommendation for sleep time, exceeding the maximum recommended screen time by almost an hour. On this occasion, there are positive, slight and significant relationships between physical activity and screen time, and a moderate relationship between screen time and sleep time.

## Figures and Tables

**Table 1:** Descriptive characteristics of the sample.

		*n* (Percentage)
Sex	Male	345 (52)
	Female	318 (48)
Grade	1st	161 (24.3)
	2nd	177 (26.7)
	3rd	154 (23.2)
	4th	171 (25.8)
Total		694 (100%)

**Table 2:** Descriptive statistics of the research variables according to sex and grade.

Variable	Total	Sex		Grade			
		Male	Female	1st	2nd	3rd	4th
MVPA	67.99 ± 43.51	79.31 ± 45.30	55.71 ± 37.88	77.92 ± 47.06	75.61 ± 47.13	59.83 ± 35.58	58.11 ± 39.18
ScT	112.56 ± 65.80	129.46 ± 67.39	94.22 ± 58.92	103.82 ± 62.41	117.76 ± 65.61	105.22 ± 57.99	122 ± 73.96
SlT	548.63 ± 50.21	552.82 ± 48.58	554.09 ± 51.61	567.82 ± 46.64	556.03 ± 46.81	541.71 ± 48.38	529 ± 50.61

Note. The units of the variables are expressed in minutes/day; Moderate–vigorous Physical Activity (MVPA); Screen Time (ScT); Sleeping Time (SlT).

**Table 3:** Descriptive statistics and z-score correlations of the research variables.

	M (SD)	CI 95%	Kolmogorov–Smirnov	Skewness	Kurtosis	2	3
1. MVPA	−0.028 ± 0.934	−0.099/0.043	0.082 *	0.823	0.227	−0.145 **	−0.050
2. Screen Time	−0.067 ± 0.858	−0.132/−0.001	0.082 *	0.851	0.533	-	0.008
3. Sleep Time	0.056 ± 0.881	−0.011/0.123	0.021	−0.112	−0.011	-	-

Note. Moderate–vigorous Physical Activity (MVPA); Mean (M); Standard Deviation (SD); Confidence interval (CI); * *p* ≤ 0.05; ** *p* ≤ 0.01.

**Table 4:** Post-hoc multiple comparisons of variables.

	Grade (I)	Grade (J)	Mean Difference (I-J)	Error Deviation	Sig.	CI (95%)	
						Lower Limit	Upper Limit
MVPA	1	2	2.31	4.65	0.96	−9.65	14.28
	1	3	18.09	4.81	*	5.71	30.48
	1	4	19.81	4.69	*	7.74	31.88
	2	3	15.78	4.70	0.01	3.67	27.89
	2	4	17.49	4.58	*	5.71	29.28
	3	4	1.72	4.74	0.98	−10.49	13.93
ScT	1	2	−13.94	7.13	0.21	−32.31	4.43
	1	3	−1.40	7.38	1	−20.40	17.61
	1	4	−18.18	7.19	0.06	−36.70	0.38
	2	3	12.54	7.22	0.30	−6.04	31.13
	2	4	−4.24	7.02	0.93	−22.32	13.84
	3	4	−16.79	7.27	0.10	−35.52	1.95

Note. Moderate–vigorous Physical Activity (MVPA); Screen Time (ScT); Confidence interval (CI); * *p* ≤ 0.001.

**Table 5:** Descriptive statistics for the three clusters.

	N (%)	Sex (%)		Grade (%)				Cluster Center (Z-Cores)			M ± SD (Minutes/Day)		
		Male	Female	1st	2nd	3rd	4th	MVPA	ScT	SlT	MVPA	ScT	SlT
Cluster 1	258 (38.91)	45	55	28.7	27.1	25.6	18.6	1.33	−0.13	2.80	48.52 ± 26.43	65.26 ± 57.69	583.73 ± 37.00
Cluster 2	187 (28.21)	61	39	30.5	29.4	17.6	22.5	2.89	−1.49	−1.52	119.41 ± 34.56	80.49 ±43.48	532.28 ± 45.73
Cluster 3	218 (32.88)	52.8	47.2	13.8	23.9	25.2	37.2	−1.06	2.95	−0.36	46.93 ± 27.36	169.39 ± 67.37	521.13 ± 42.67

Note. Moderate–vigorous Physical Activity (MVPA). Screen time (ScT); Sleeping Time (SlT).

**Table 6:** Post-hoc multiple comparisons of variables in clusters.

	Grade (I)	Grade (J)	Mean Difference (I-J)	Error Deviation	Sig.	CI (95%)	
						Lower Limit	Lower Limit
MVPA	1	2	−1.52	0.06	*	−1.66	−1.38
	1	3	0.03	0.06	0.83	−0.10	0.17
	2	3	1.56	0.06	*	1.41	1.70
ScT	1	2	0.10	0.07	0.32	−0.06	0.25
	1	3	−1.06	0.06	*	−1.21	−0.92
	2	3	−1.16	0.07	*	−1.32	−0.99
SlT	1	2	0.90	0.07	*	0.74	1.07
	1	3	1.10	0.07	*	0.94	1.26
	2	3	0.20	0.07	0.02	0.03	0.37

Note. Moderate–Vigorous Physical Activity (MVPA); Screen Time (ScT); Sleep Time (SlT); Confidence interval (CI); * *p* ≤ 0.001.

**Table 7:** Descriptive statistics and correlations between variables in each cluster.

		Kolmogorov–Smirnov	Skewness	Kurtosis	2	3
Cluster 1	MVPA	0.091 *	0.463	−0.530	0.123 *	0.214 **
	ScT	0.072 *	0.348	−0.432	-	0.288 **
	SlT	0.029	0.263	0.302	-	-
Cluster 2	MVPA	0.084 *	0.546	−0.208	0.172 *	0.324 **
	ScT	0.064	0.435	−0.545	-	0.166 *
	SlT	0.033	0.013	0.200	-	-
Cluster 3	MVPA	0.074 *	0.781	0.641	0.168 **	0.005
	ScT	0.050	0.267	−0.354	-	0.470 **
	SlT	0.052	−0.211	−0.049	-	-

Note: Moderate–Vigorous Physical Activity (MVPA); Screen Time (ScT); Sleep Time (SlT); ** *p* ≤ 0.01; * *p* ≤ 0.05.

## Data Availability

Not applicable.
